# Immune Response Elicited by DNA Vaccination Using *Lactococcus lactis* Is Modified by the Production of Surface Exposed Pathogenic Protein

**DOI:** 10.1371/journal.pone.0084509

**Published:** 2014-01-21

**Authors:** Daniela Pontes, Marcela Azevedo, Silvia Innocentin, Sébastien Blugeon, François Lefévre, Vasco Azevedo, Anderson Miyoshi, Pascal Courtin, Marie-Pierre Chapot-Chartier, Philippe Langella, Jean-Marc Chatel

**Affiliations:** 1 State University of Paraiba, Campus V, Department of Biological Sciences. João Pessoa, Paraíba, Brazil; 2 INRA, UMR1319 Micalis, Domaine de Vilvert, Jouy-en-Josas, France; 3 AgroParisTech, UMR1319 Micalis, Jouy-en-Josas, France; 4 Institute of Biological Sciences, Federal University of Minas Gerais (UFMG-ICB), Belo Horizonte, Minas Gerais, Brazil; 5 Lymphocyte Signaling and Development Laboratory, Babraham Institute, Babraham Research Campus, Cambridge, United Kingdom; 6 INRA, VIM, Domaine de Vilvert, Jouy-en-Josas, France; University Paris South, France

## Abstract

In this study, we compared immune responses elicited by DNA immunization using *Lactococcus lactis* or *L. lactis* expressing the *Staphylococcus aureus* invasin Fibronectin Binding Protein A (FnBPA) at its surface. Both strains carried pValac:BLG, a plasmid containing the cDNA of Beta-Lactoglobulin (BLG), and were designated LL-BLG and LL-FnBPA+ BLG respectively. A T_H_2 immune response characterized by the secretion of IL-4 and IL-5 in medium of BLG reactivated splenocytes was detected after either oral or intranasal administration of LL-FnBPA+ BLG. In contrast, intranasal administration of LL-BLG elicited a T_H_1 immune response. After BLG sensitization, mice previously intranasally administered with LL-BLG showed a significantly lower concentration of BLG-specific IgE than the mice non-administered. Altenatively administration of LL-FnBPA+ BLG didn't modify the BLG-specific IgE concentration obtained after sensitization, thus confirming the T_H_2 orientation of the immune response. To determine if the T_H_2-skewed immune response obtained with LL-FnBpA+ BLG was FnBPA-specific or not, mice received another *L. lactis* strain producing a mutated form of the *Listeria monocytogenes* invasin Internalin A intranasally, allowing thus the binding to murine E-cadherin, and containing pValac:BLG (LL-mInlA+ BLG). As with LL-FnBPA+ BLG, LL-mInlA+ BLG was not able to elicit a T_H_1 immune response. Furthermore, we observed that these difference were not due to the peptidoglycan composition of the cell wall as LL-FnBPA+ BLG, LL-mInlA+ BLG and LL-BLG strains shared a similar composition. DNA vaccination using LL-BLG elicited a pro-inflammatory T_H_1 immune response while using LL-FnBPA+ BLG or LL-mInlA+ BLG elicited an anti-inflammatory T_H_2 immune response.

## Introduction

An innovative strategy using *Lactococus lactis,* a food grade bacterium, to deliver plasmids *in vitro* [1] and *in vivo* [2] was previously developed by our laboratory. We first demonstrated that a non invasive, transiting bacterium was capable of transferring a plasmid to the epithelial membrane of the small intestine in mice and eliciting a Type 1 T helper cell (T_H_1) immune response [2]. A T_H_1 type of immune response is typical for DNA vaccination [3] regardless of various factors which can influence the immune response [4], such as the nature of the antigen or mouse strain. To better understand the mechanism of plasmid transfer and thus possibly improve on it, we developed invasive *L. lactis* strains by expressing invasins on their cell surface.

We previously constructed LL-FnBPA+, a recombinant *L. lactis* strain expressing *Staphylococcus aureus* Fibronectin Binding protein A (FnBPA) on its surface and demonstrated its potential for plasmid DNA delivery *in vitro* [5]. More recently, we confirmed that the use of invasive LL-FnBPA+ increases plasmid transfer rate/efficacy *in vivo* [6]. In addition, a recombinant *L. lactis* strain producing *Listeria monocytogenes* Internalin A (LL-InlA+) was constructed and demonstrated to be capablefor plasmid transfer *in vivo* with guinea pigs [7]. As InlA binds only poorly to murine E-cadherin, the use of this strain is thereby limited to either transgenic mice expressing human E-cadherin [8] or guinea pigs. InlA has been mutated to increase its binding to murine E-cadherin [9]. We produced mutated mInlA at the surface of *L. lactis* (LL-mInlA+) and showed that LL-mInlA+ did not significantly increase plasmid transfer rate in mice [10].

In this manuscript, we evaluated wether the immune response elicited by *L. lactis*-mediated DNA immunization could be modified by production of invasins on the cell surface. We report that intranasal or oral DNA administration using invasive LL-FnBPA+ carrying pValacBLG (LL-FnBPA+ BLG) elicits a T_H_2 primary immune response whereas the non invasive strain (LL-BLG) elicited a classical T_H_1 immune response. These results were confirmed using another *L. lactis* strain producing a mutated InlA and carrying pValacBLG (LL-mInlA+ BLG). We reported previously that oral administration of LL-mInlA+ BLG or LL-FnBPA+ BLG will elicit the production of BLG in mice enterocytes [10], [11].Differences between immune responses observed after mucosal administration of invasive and non invasive strains were not due to a difference in peptidoglycan composition. Unexpectedly production of two unrelated different invasins from pathogens led to the similar modification of the intrinsic immunomodulatory properties of *L. lactis* with respect to DNA delivery.

## Results

### DNA immunization using LL-FnBPA+ BLG or LL-BLG strains elicits different BLG-specific primary immune responses

**Figure 1:**
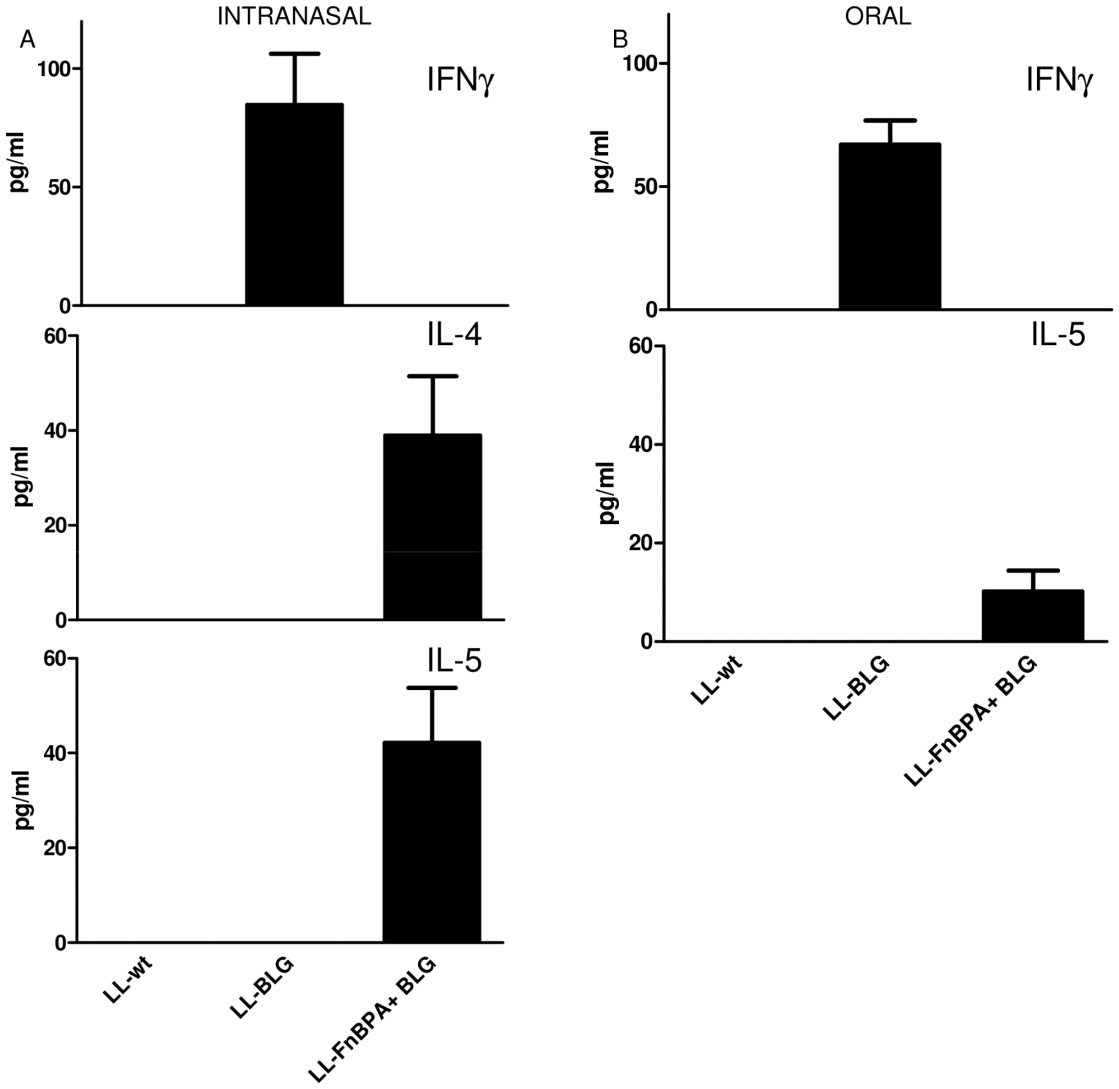
BLG-specific cytokines secreted by BLG-reactivated splenocytes from mice orally or intranasally administered with LL-wt, LL-BLG or LL-FnBPA+ BLG. Mice were intranasally (A) or orally (B) administered with LL-wt, LL-BLG or LL-FnBPA+ BLG then splenocytes from pooled samples were purified and reactivated with BLG. Secreted cytokines, IFN-γ, IL-4 and IL-5 were assayed in medium. Sum of two independent experiments, 8 mice/group.

In order to know if the production of FnBPA at the surface of *L. lactis* could influence its immunomodulatory properties, mice were orally or intranasally administered with LL-wt (control), LL-BLG or LL-FnBPA+ BLG strains and the BLG specific primary immune response was monitored. No BLG specific IgG1, IgG2a or IgE could be detected in mice sera regardless of administration route (data not shown). Splenocytes from orally and intranasally administered mice were reactivated with BLG and secreted IFN-γ, IL-4 and IL-5 cytokines were assayed in the medium. Splenocytes of mice intranasally (Fig. 1 A) or orally immunized (Fig. 1 B) with LL- BLG strain secreted only IFN- γ, IL-4 and IL-5 were detected in medium of BLG-reactivated splenocytes from mice receiving LL-FnBPA+ BLG (Fig. 1 A) intranasally. Moreover, only IL-5 cytokine secretion by BLG-reactivated splenocytes from mice orally immunized with LL-FnBPA+ BLG was observed (Fig. 1 B). No IL-4 secretion was detected in mice orally administered with LL-FnBPA+ BLG (data not shown). Therefore, mice treated with LL-FnBPA+ BLG exhibit a T_H_2 BLG-specific immune response, whereas a classical T_H_1 BLG-specific immune response was observed in mice treated with LL-BLG.

### Mice undergoing intranasal administration of LL-BLG were protected from further BLG sensitization in contrast to mice pre-administered with LL-FnBPA+ BLG

**Figure 2:**
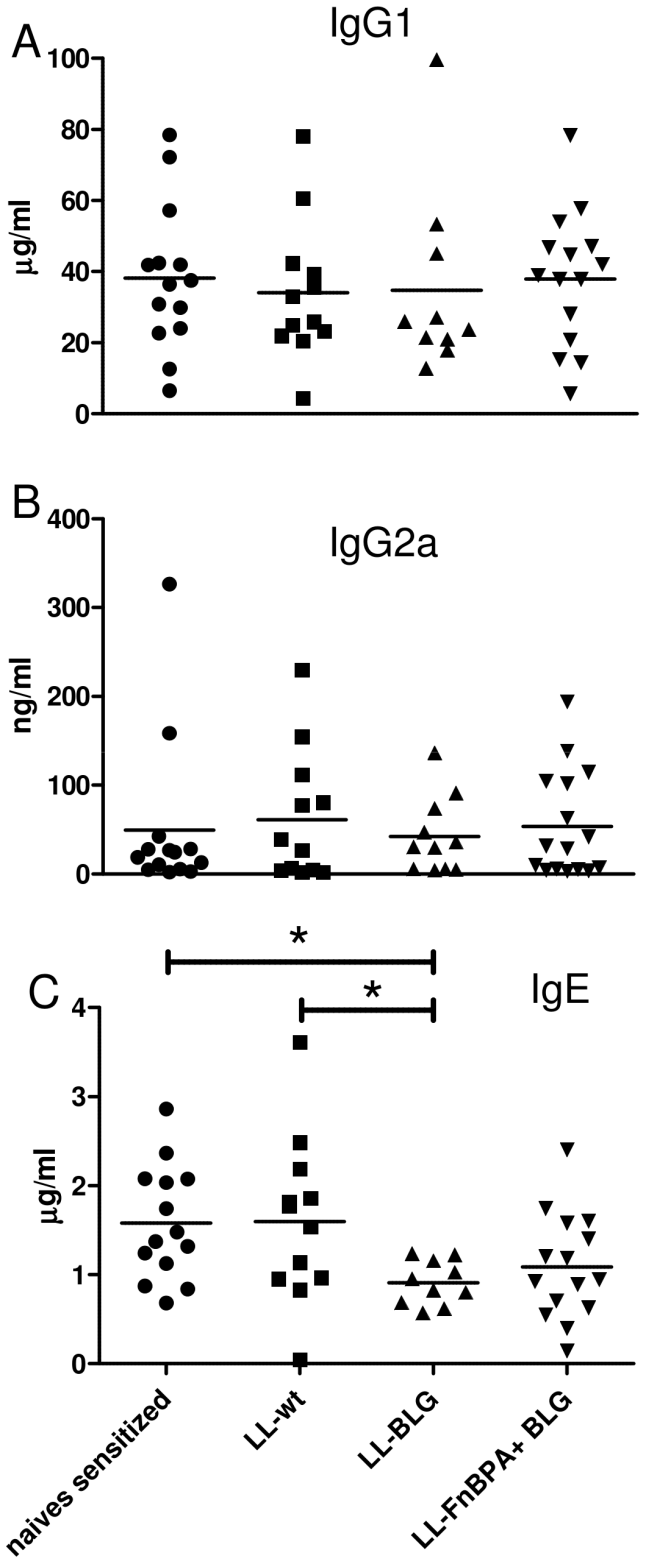
BLG-specific IgG1, IgG2a and IgE in sera of mice intranasally administered with LL-wt, LL-BLG or LL-FnBPA+ BLG and then sensitized with BLG. BLG specific IgG1 (A), IgG2a (B) and IgE (C) were assayed in sera of mice intranasally administered or not (naive-sensitized, NS) with LL-wt, LL-BLG or LL-FnBPA+ BLG then sensitized with BLG in Alum in order to elicit a Th2 immune response. Sum of two independent experiments.* indicates P< 0.05, ANOVA and Tukey's post test.

After intranasal or oral administration of LL-wt, LL-BLG or LL-FnBPA+ BLG strains, mice were sensitized with BLG in alum in order to elicit a T_H_2 immune response. The concentrations of BLG-specific IgG1, IgG2a and IgE were monitored in sera of sensitized and intranasally pre-treated mice (Fig.2). Naïve sensitized mice (mice not receiving bacteria) exhibited a T_H_2 immune response characterized by a high level of BLG-specific IgG1 and IgE. Pre-treatment with LL-wt control strain had no effect on IgG1, IgG2a or IgE level. Despite a decrease of 25% on average, there was no significant difference in IgE level between naive sensitized mice and mice pre-treated with LL-FnBPA+ BLG (Fig. 2). In contrast, mice pre-treated with LL-BLG showed a statistically significant decrease of 45% less BLG-specific IgE antibody (Fig. 2).

No differences in the levels of IgG1, IgG2a or IgE could be detected between mice orally immunized with LL-wt, LL-BLG or LL-FnBPA+ BLG strains and naive sensitized mice (data not shown).

### Cytokines secreted by BLG reactivated splenocytes after BLG sensitization confirmed the T_H_2 orientation of the immune response elicited by the administration of LL-FnBPA+ BLG

**Figure 3:**
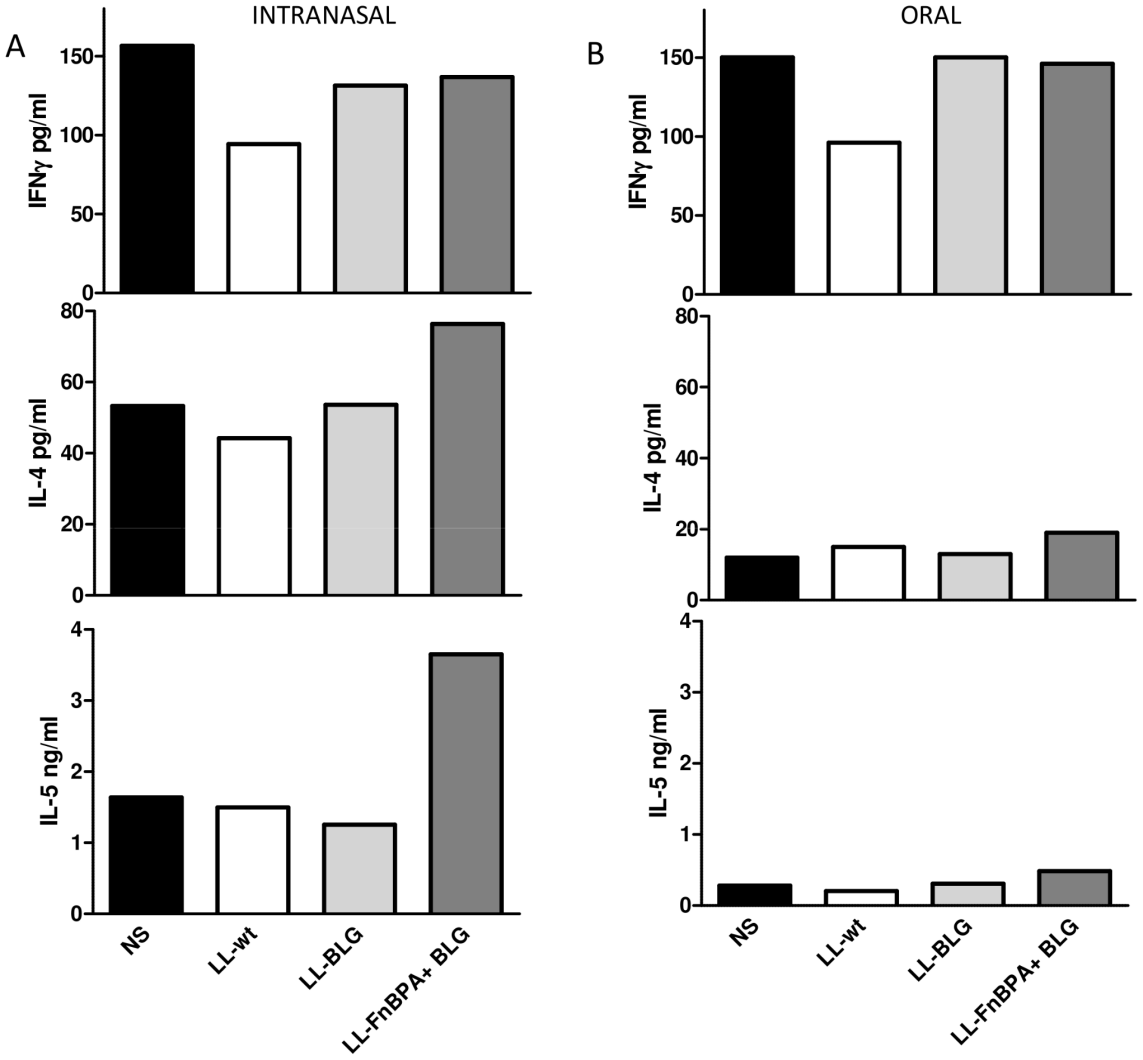
BLG-specific cytokines secreted by BLG-reactivated splenocytes from mice orally or intranasally administered with LL-wt, LL-BLG or LL-FnBPA+ BLG then BLG sensitized. Mice were intranasally (A) or orally (B) administered or not (NS) with LL-wt, LL-BLG or LL-FnBPA+ BLG then sensitized with BLG in Alum. Splenocytes from pooled samples were purified and reactivated with BLG. Secreted cytokines, IFN-γ, IL-4 and IL-5 were assayed in medium. The results presented here are from one experiment representative of two performed independently, 8 mice/group.

Splenocytes of mice sensitized after intranasal or oral pre-treatment with either LL-FnBPA+ BLG or LL-BLG strains were reactivated by BLG and IFN- γ, IL-4 and IL-5 cytokines were assayed in medium (Fig. 3). Regardless of the route of administration, mice pre-treated with LL-wt control strain exhibited a lower level of IFN- γ and IL-5 as compared to the naïve-sensitized group. Therefore, a slight increase of IL-4 levels (Fig. 3B) was observed only for mice orally pre-treated with LL-wt strain. Intranasal and oral pre-treatments with LL-BLG did not modify the levels of any cytokines. However, spleen cells from all sensitized mice pre-treated with LL-FnBPA+ BLG secreted higher amounts of IL-4 and IL-5 than those of the naïve-sensitized group (Fig. 3).

### Intranasal administration of invasive LL-mInlA+ BLG does not elicit a T_H_1 BLG-specific primary immune response

**Figure 4:**
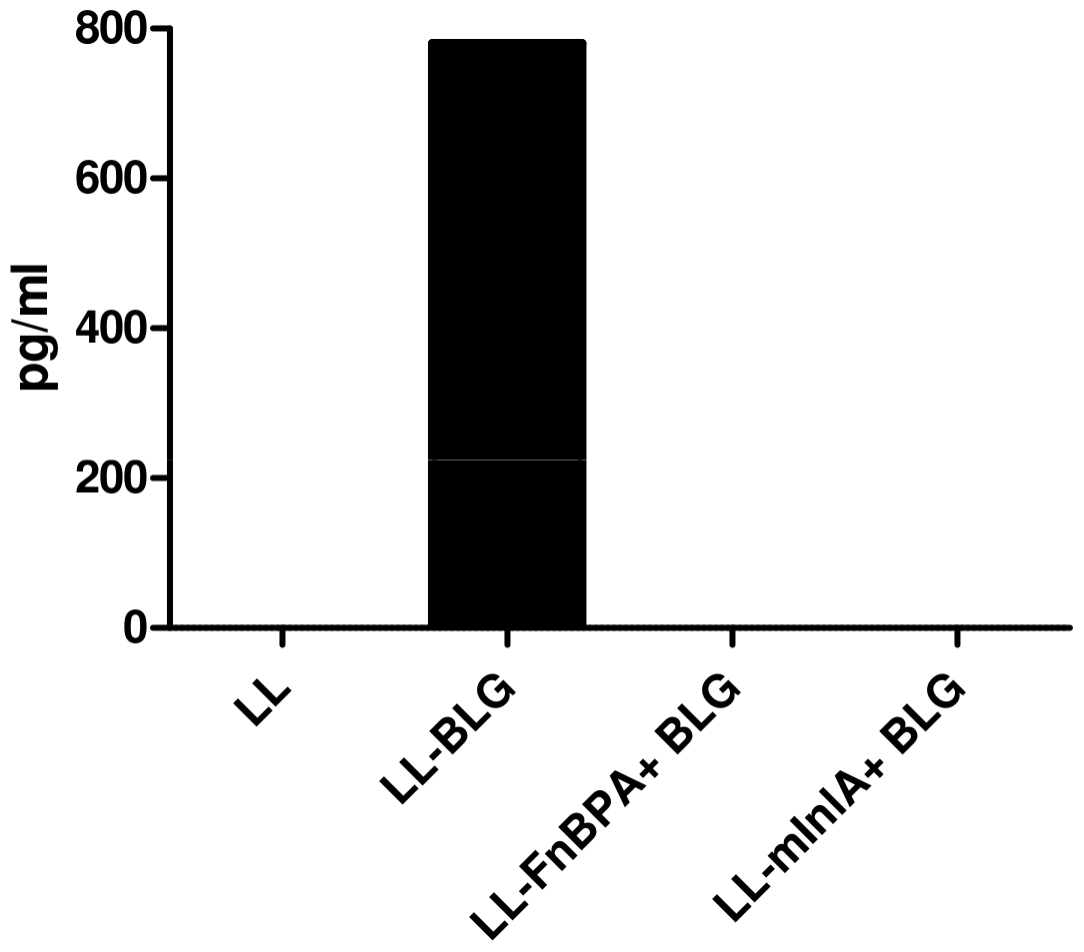
BLG-specific cytokines secreted by BLG-reactivated splenocytes from mice intranasally administered with LL-wt, LL-BLG, LL-FnBPA+ BLG or LL-mInlA+ BLG. Mice were intranasally administered with LL-wt, LL-BLG, LL-FnBPA+ BLG or LL-mInlA+ BLG then splenocytes from pooled samples were purified and reactivated with BLG. Secreted IFN-γ was assayed in medium.

To study if the T_H_2 orientation of the immune response was or not FnBPA-specific, another invasive strain, LL-mInLA+ BLG, was administered intranasally to mice. Splenocytes from mice pre-treated with LL-BLG, LL-FnBPA+ BLG or LL-mInlA+ BLG were reactivated with BLG and IFN- γ, IL-4 and IL-5 were assayed in the medium. Splenocytes of mice pre-treated with non invasive LL-BLG secreted only IFN- γ (Fig. 4), whereas no cytokines were detected in media of mice pre-treated with both invasive LL-FnBPA+ BLG or LL-mInlA+ BLG strains.

### Peptidoglycan composition of cell wall from LL-BLG, LL-FnBPA+ BLG and LL-mInLA+ BLG is highly similar

**Figure 5:**
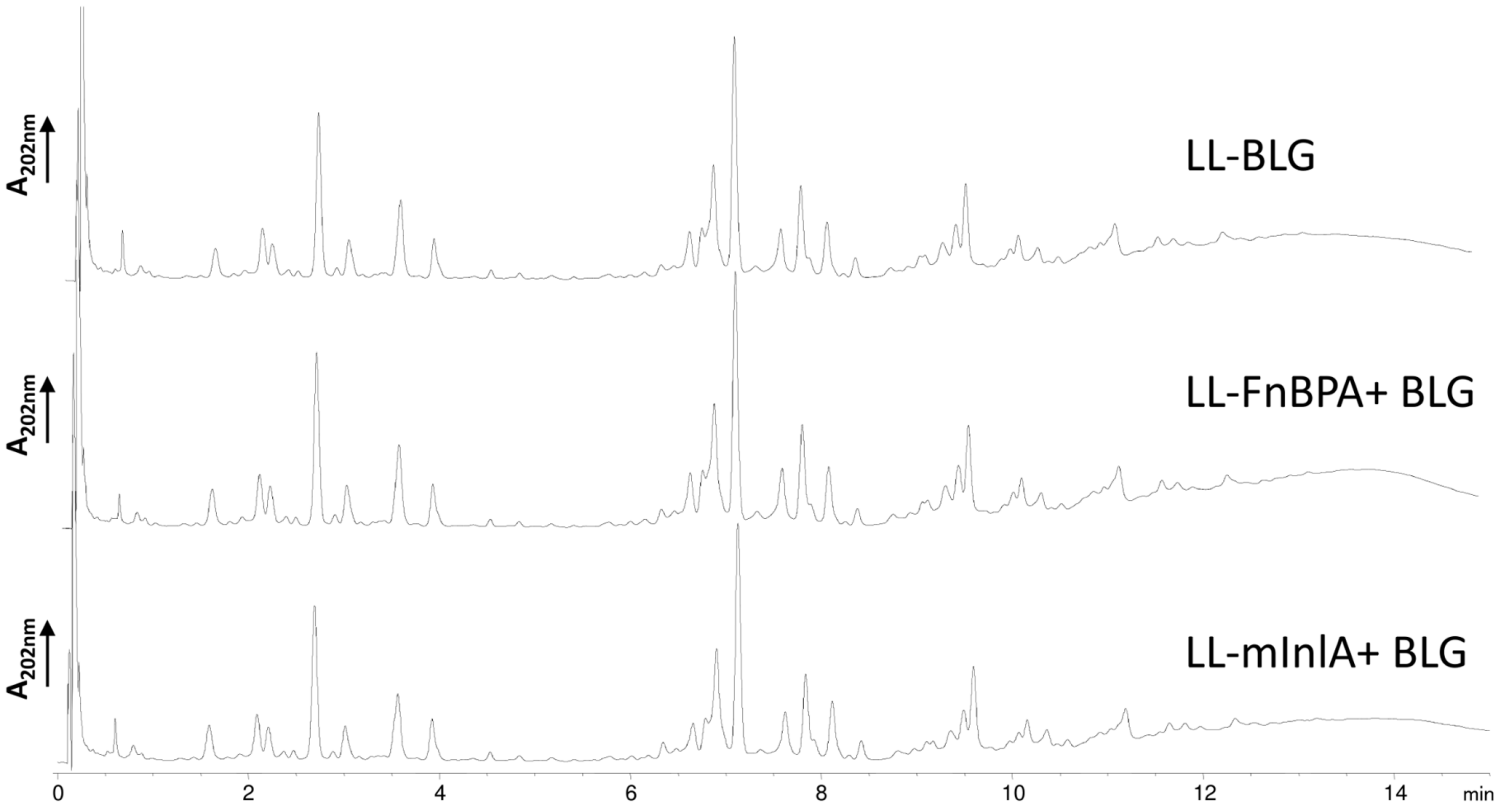
Peptidoglycan composition of cell wall from lactococci recombinant strains. The PG structure of the different strains LL-BLG, LL-FnBPA+ BLG and LL-mInlA+ BLG was compared. PG was extracted from each strain, digested with mutanolysin and the resulting muropeptides were separated by RP-HPLC. Absorbance was monitored at 220 nm.

Peptidoglycan (PG) of cell wall is well known for its adjuvanticity role. We studied if the production of heterologous proteins as invasins at the surface of *L. lactis* would modify its composition. The PG composition of the different strains LL-BLG, LL-FnBPA+ BLG and LL-mInlA+ BLG was compared. For this purpose, PG was extracted from each strain, digested with mutanolysin and the resulting muropeptides were separated by RP-HPLC. Comparison of the obtained muropeptide profiles (Fig. 5) did not reveal any differences at the level of PG composition between the strains.

## Discussion

In this paper, we compared the immune response induced in mice via DNA immunization by administration of either non invasive or recombinant *L. lactis* strain producing FnBPA or mInLA. We previously demonstrated *in vitro* and *in vivo* that *L.lactis* is able to transfer a fully functional plasmid to eukaryotic cells of the murine epithelial membrane [1], [2]. In order to understand and improve the DNA delivery, recombinant strains producing invasins from various pathogens, such as FnBPA, InlA, or a mutated form of InlA, were developed. *In vitro*, all of our invasive strains were better DNA delivery vectors than non invasive strains [7], [11], [18]. *In vivo,* the use of the invasive LL-FnBPA+ BLG strain increased the number of mice producing BLG, but not the quantity of BLG produced [11] whereas the use of LL-mInlA+ BLG did not significantly change either of the two aforementioned parameters [18]. Herein, we would like to determine the incidence of the invasin expression on the nature of the immune response elicited by administration of such strains compared to non invasive ones.

Since 1996 it was clearly shown that T_H_1 is the main immune response elicited by DNA immunization [3]. Several previous approaches had demonstrated that the nature of the antigen [4], [19], [20], the route of administration [21], [22], [23] or the genetic background of the host [4], [24] could significantly influence the immune response.

We previously published that DNA vaccination using non-invasive lactococci elicits a T_H_1 type of immune response [2]. In this study, we confirmed this result and report for the first time on intranasal DNA immunization, using recombinant invasive lactococci strains. After intranasal administration, the wild type LL-BLG strain induced a T_H_1 type of immune response as represented by the presence of IFN-γ cytokine secretion. We compared the immune responses observed with LL-BLG using different routes of administration and showed that intranasal immunization was more effective than oral immunization. Similar results have already been described for protein vaccination using Lactic Acid Bacteria (LAB) [25], [26].

Surprisingly, intranasal or oral administration of LL-FnBPA+ BLG strain elicited a T_H_2 type of immune response characterized by secretion of IL-4 or IL-5 by BLG reactivated splenocytes. After BLG sensitization, no decrease of IgE concentration was observed in mice. The results clearly showed that DNA immunization using invasive LL-FnBPA+ BLG strain polarized the immune response toward a T_H_2-type in contrast to the T_H_1-type response elicited by DNA vaccination using non invasive LL-BLG strain. We explored if this phenomenon was FnBPA-specific or if it was linked to the invasivity properties of our strain. In order to answer to this question, we performed the same type of experiments using a new recombinant invasive strain producing a mutated form of Internalin A from *Listeria monocytogenes*, LL-mInlA+ BLG. Invasivity of LL-FnBPA+ BLG and LL-mInlA+ BLG were tested *in vitro* and were comparable [18]. *In vivo* BLG expression detected in enterocytes was comparable between mice orally administered with LL-BLG, LL-FnBPA+ BLG or LL-mInlA+ BLG (data not shown). We were not able to detect any IFN-γ in medium of BLG reactivated splenocytes from mice treated with LL-mInlA+ BLG. Moreover, neither IL-4 nor IL-5 was detected but the level of IFN-γ secreted by BLG reactivated splenocytes from mice administered with LL-BLG was 10-times higher than in the previous experiments described in this paper. This suggests that the production of both FnBPA and mInlA invasins at the surface of *L. lactis* skewed the immune response towards a T_H_2 instead of T_H_1 type of immune response.

It has been recently described that production of invasins at the surface of lactobacilli can modify their immunomodulatory properties. The production of invasin from *Yersinia pseudotuberculosis* at the surface of *Lactobacillus plantarum* changed its profile from neutral to pro-inflammatory [27]. The authors determined the immunomodulatory properties of their strain only *in vitro* using monocytes stably transfected with NF-κB reporter system.

Usually, bacteria used as DNA delivery vectors are attenuated pathogens like *L. monocytogenes* or recombinant invasive *E. coli* [28], [29]. *L. monocytogenes* infection is accompanied by a strong innate immune response followed by a T-cell activation of T_H_1 type [30]. Attenuated *Salmonella typhimurium* delivering a DNA vaccine coding for cyst wall protein-2 (CWP-2) from *Giardia lamblia* was able to elicit a mixed CWP-2 specific cellular T_H_1/T_H_2 immune response after oral administration [31]. A humoral immune response was characterized by the presence of CWP-2 specific IgG2a in sera. Moreover, the use of invasive *E. coli* expressing the invasin from *Y. pseudotuberculosis* and Listeriolysin-O (LLO) from *L. monocytogenes* could elicit a T_H_1 immune response characterized by secretion of IFNγ from reactivated splenocytes. Similar results were obtained when non invasive *E. coli* was used as DNA delivery vector, but the induction of immune response was less effective. In addition no T_H_2 cytokines were assayed. Protection against challenge could be induced with a better efficiency than naked DNA [32]. Using the same type of bacterial vector to deliver GFP, Harms et al. [33] observed a mixed T_H_1/T_H_2 immune response for both invasive and non invasive *E. coli*. The secondary immune response provides a more defining T_H_1 cytokine profile. Our results with DNA vaccination using non-invasive lactococci elicited this "classical" T_H_1 immune response, while the use of LL-FnBPA+ BLG or LL-mInlA+ BLG invasive strains led to a T_H_2 type immune response. However the immune response elicited by the use of invasive *L. lactis* was low level. It is possible that the main immune response was a tolerization characterized by a high number of Treg.

We know that invasive or non invasive *L. lactis* strains can transfer plasmids *in vivo,* to enterocytes [11], but other subsets of epithelial cells may also be targeted by the invasive LAB, thus modifying the immune response. Tropism of non invasive *E. coli* and invasive *E. coli* expressing the invasin from *Y. pseudotuberculosis*, LLO from *L. monocytogenes* or GFP protein at the cell surface were observed *ex vivo* in murine small intestine tissue. Invasive *E. coli* was mainly detected in the Peyer's patches. Non invasive *E. coli* can gain also entry to the Peyer's patches, but with less efficiency [34]. Invasive bacteria were also assayed in Peyer's patch antigen-presenting cells. 3% of the DCs and 0.5% of the leukocytes were GFP positive [34]. The same phenomenon could happen with our recombinant lactococci and might explain the differences between the type of immune responses. If the invasive lactococci preferentially penetrate into the Peyer patches, BLG produced in M cells and phagocytosed by macrophages would then be presented under a MHC II context, eliciting a CD4+T_H_2 cell immune response characterized by the secretion of IL-4 or IL-5 as seen in our experiments. Alternately, non invasive lactococci would transfer plasmids mostly to enterocytes or DCs which will then present BLG under a MHC I context eliciting a CD8+ T cells immune response characterized by the secretion of IFNγ. Currently, there is no supporting evidence available to explain why invasive lactococci could penetrate with more efficiency in M cells than in enterocytes.

E-cadherin, mInlA receptor, is expressed on the basolateral membrane of epithelial cells which are strongly linked to each other in the gut turning E-cadherin less available. It has been shown recently that *L. monocytogenes* could enter in the epithelial membrane through extruding epithelial cells at the top of the villi but mainly through goblet cells which are located deeper in the crypt [35].

The difference of immune response between invasive and non invasive lactococci could also be explained by a difference in the composition of the cell wall. Indeed some recent studies have revealed the crucial role of cell wall components in immune response against bacteria like *Bacteroides fragilis* [36], [37] or probiotics [38], [39]. Thus, we decided to check if the expression of invasins at the surface of *L. lactis* could modify the peptidoglycan composition of our recombinant strain, but no differences were detected.

To better understand why *L. lactis* recombinant invasive strains produced a low T_H_2 response, we need to go further in the characterization of the immune response, for example the subset of T cell involved, the nature of cells taken up our bacteria or plasmid. It would be interesting to know if the same bias is observed when we use our invasive bacteria to deliver proteins instead of plasmid. The data described here will help us to choose the vehicle depending on the nature of the desired immune response, pro-inflammatory or anti-inflammatory, to be induced by DNA delivery.

## Materials and Methods

### Ethics statement

All procedures were carried out in accordance with European and French guidelines for the care and use of laboratory animals. Permission 78–123 is a permit number dedicated to P. Langella. MICALIS (Microbiologie de l'Alimentation au Service de la Santé) review board specifically approved this study.

### Bacterial strains, plasmids, media and growth conditions

Bacterial strains and plasmids used in this study are listed in Table 1. *L. lactis* subsp. *cremoris* strains were grown in M17 medium containing 0.5% glucose (GM17) at 30°C. *E. coli* strains were grown in Luria–Bertani medium and incubated at 37°C with vigorous shaking. Antibiotics were added at the indicated concentrations as necessary: erythromycin, 500 µg/ml for *E. coli*, and 5 µg/ml for *L. lactis*; chloramphenicol, 10 µg/ml for *E. coli* and *L. lactis*.

**Table 1:** Plasmids and strains used in this study.

Strains	Properties	Reference
**LL-wt**	MG1363 *L. lactis* strain carrying pIL253 plasmid, Ery^r^	[40]
**LL- BLG**	MG1363 *L. lactis* strain carrying pIL253 and pValac:BLG plasmid, Ery^r^, Cm^r^	[11]
**LL-FnBPA+ BLG**	MG1363 *L. lactis* strain expressing FnBPA and carrying pValac:BLG, Ery^r^, Cm^r^	[11]
**LL-mInlA+ BLG**	NZ9000 *L. lactis* strain expressing mInlA and carrying pValac:BLG, Ery^r^, Cm^r^	[10]

Ery^r^, Erythromycin; Cm^r^, Chloramphenicol

### Mice handling

Specific pathogen-free BALB/c mice (females, 6 weeks of age; Janvier, France) were maintained under normal husbandry conditions in the animal facilities of the National Institute of Agricultural Research (UEAR, INRA, Jouy-en-Josas, France). All animal experiments began after the animals were allowed 2 weeks of acclimation and were performed according to European Community rules of animal care and with authorization 78–149 of the French Veterinary Services.

### Apparatus and reagents

All enzymatic immunoassays were performed in 96-well microtitre plates (Immunoplate Maxisorb, Nunc, Roskilde, Denmark) using specialized Titertek microtitration equipment from Labsystems (Helsinki, Finland). Unless otherwise stated, all reagents were of analytical grade from Sigma (St Louis, MO, USA). BLG was purified from cow's milk as previously described [12].

### Quantification of BLG-specific IgE, IgG1 and IgG2a

Blood samples were obtained from the retro-orbital venous plexus, centrifuged, supplemented with 0.1% sodium azide as a preservative, and the sera was stored at -20 °C until further assay. Naïve mice were bled on the same days to assess non-specific binding. Each immunization group was composed of 8 mice. BLG- specific IgE, IgG1 and IgG2a were measured using immunoassays, as previously described, allowing quantification of antibodies recognizing both native and denatured BLG [13]. Quantification of specific IgE is preceded by removal of serum IgG using protein G immobilized on porous glass (PROSEP, Bioprocessing, Consett, UK) avoiding IgG interference in IgE detection [14]. The minimum detectable concentrations are 8 pg/ml for IgE, 7 pg/ml for IgG1 and 12 pg/ml for IgG2a.

### Cytokine production

Mice were humanely killed and spleens were harvested under sterile conditions, and pooled per immunization group. After lysis of red blood cells (180 mM NH_4_Cl, 17 mM Na_2_EDTA) and several washes, splenocytes were resuspended in RPMI-10 (RPMI supplemented with 10% foetal calf serum, 2 mM L-glutamine, 100 U penicillin, 100 mg/mL streptomycin). Cells were incubated for 60 h at 37°C (5% CO2) in 96-well culture plates (10^6^ cells/well) in the presence of BLG (20 µg/mL) or concanavalin A (1 µg/mL, positive control). Incubations with PBS or ovalbumin (20 µg/mL) were done as negative controls. Supernatants were then removed and stored at -80°C until further assay. IFN-γ, IL-4 and IL-5 was assayed using CytoSetsTM kits (BioSource International Europe, Nivelles, Belgium). Limits of detection are respectively of 5 pg/ml, 3 pg/ml and 1 pg/ml for IL-4, IL-5 and IFN-γ.

### Primary immune response elicited by oral or intranasal administration of bacteria strains

Strains were grown to saturation (overnight (ON) cultures) as described above. Before administration, strains were centrifuged at 5,000 g for 10 min at 4°C, and then resuspended in PBS to wash the bacteria. Washed bacteria were centrifuged again and the pellet was resuspended in PBS containing 10% Fetal Calf Serum to provide fibronectin during 2 hours of incubation at 4°C [11]. Then the strains were pelleted and resuspended in PBS. Groups of mice (n = 8) were fed orally with 10^9^ CFU/mouse or received 10^9^ CFU in 10 µl per nostril intranasally over 3 consecutive days. Two weeks after the last administration, mice received a booster, with 3 consecutive days of immunization with10^9^ CFU/mouse by oral administration or 10^9^ CFU by intranasal administration. One week after the last administration, mice were killed and bled. Naïve mice were killed and bled the same day to assess non-specific immune response.

### Immune response after sensitization in mice intranasally or orally administered with bacteria strains

Mice were immunized following the same protocol listed in the aforementioned section. Two weeks after the final booster administration, all mice were sensitized by intraperitoneal injection of 5 µg BLG adsorbed on alum (1 mg/mouse; Alhydrogel 3%, Superfos Biosector als, Denmark) [15]. Mice were injected with a volume of 0.2 ml per mouse. Two weeks post-sensitization, mice were humanely killed and bled. Serum was collected for assessing BLG-specific IgE, IgG1 and IgG2a concentrations as described above. Naïve mice (n = 8) were bled the same day to assess non-specific immune response. Naive sensitized mice received PBS orally or intranasally instead of bacteria. Spleens were harvested and isolated splenocytes were reactivated for cytokines secretion as described above.

### Peptidoglycan structural analysis

Peptidoglycan was extracted from exponential phase cultures of the different *L. lactis* strains as described previously [16]. Peptidoglycan was then hydrolyzed with mutanolysin and the reduced soluble muropeptides were then separated by RP-HPLC with an Agilent UHPLC1290 system using ammonium phosphate buffer and methanol linear gradient as described previously [17].

### Statistical analyses

All statistical calculations were done using GraphPad Prism version 5.00 for Windows (GraphPad Software, San Diego, CA). Data were analyzed using analysis of variance (ANOVA) and Tukey's multiple comparison test. A p-value less than 0.05 was considered significant.
